# The Japanese Breast Cancer Society Clinical Practice Guidelines for systemic treatment of breast cancer, 2018 edition

**DOI:** 10.1007/s12282-020-01085-0

**Published:** 2020-04-02

**Authors:** Tatsunori Shimoi, Shigenori E. Nagai, Tetsuhiro Yoshinami, Masato Takahashi, Hitoshi Arioka, Mikiya Ishihara, Yuichiro Kikawa, Kei Koizumi, Naoto Kondo, Yasuaki Sagara, Masahiro Takada, Toshimi Takano, Junji Tsurutani, Yoichi Naito, Rikiya Nakamura, Masaya Hattori, Fimikata Hara, Naoki Hayashi, Toshiro Mizuno, Minoru Miyashita, Nami Yamashita, Takashi Yamanaka, Shigehira Saji, Hiroji Iwata, Tatsuya Toyama

**Affiliations:** 1 Department of Breast and Medical Oncology, National Cancer Center Hospital, 5-1-1 Tsukiji,, Chuo-ku, Tokyo 104-0045 Japan; 2 Department of Breast Oncology, Saitama Cancer Center, 780 Komuro, Ina-machi, Kitaadachi-gun, Saitama, 362-0806 Japan; 3 Department of Breast and Endocrine Surgery, Graduate School of Medicine, Osaka University, 2-2-E 10 Yamadaoka, Suita, Osaka 565-0871 Japan; 4 Department of Breast Surgery, NHO Hokkaido Cancer Center, 4-2 Kikusui, Shiroishi-ku, Sapporo, 003-0804 Japan; 5 Department of Medical Oncology, Yokohama Rosai Hospital, 3211 Kozukue, Kohoku-ku, Yokohama, Kanagawa 222-0036 Japan; 6 Department of Medical Oncology, Mie University Hospital, 2-174 Edobashi, Tsu, Mie 514-8507 Japan; 7 Department of Breast Surgery, Kobe City Medical Center General Hospital, 2-1-1, Minatojimaminamimachi, Chuo-ku, Kobe, Hyogo 650-0047 Japan; 8 First Department of Surgery, Hamamatsu University School of Medicine, 1-20-1 Handayama, Higashi-ku, Hamamatsu City, Shizuoka 431-3192 Japan; 9 Department of Breast Surgery, Nagoya City University Graduate School of Medical Sciences, 1 Kawasumi, Mizuho-cho, Mizuho-ku, Nagoya 467-8601 Japan; 10 Department of Breast Surgical Oncology, Hakuaikai Social Cooperation, Sagara Hospital, 3-31 Matsubara-cho, Kagoshima, 892-0098 Japan; 11 Department of Breast Surgery, Kyoto University Hospital, 54 Kawaharacho, Shogoin, Sakyo-ku, Kyoto, 606-8507 Japan; 12 Department of Medical Oncology, Toranomon Hospital, 2-2-2 Toranomon, Minato-ku, Tokyo, 105-8470 Japan; 13 Department of Medical Oncology, Advanced Cancer Translational Research Institute, Showa University, 1-5-8 Hatanodai, Shinagawa, Tokyo Japan; 14 Department of Breast and Medical Oncology, National Cancer Center Hospital East, 6-5-1 Kashiwanoha, Kashiwa, Chiba 277-8577 Japan; 15 Department of Breast Surgery, Chiba Cancer Center, 666-2 Nitona-cho, Chuo-ku, Chiba, Chiba 280-8717 Japan; 16 Department of Breast Oncology, Aichi Cancer Center, 1-1 Kanokoden, Chikusa-ku, Nagoya, 464-8681 Japan; 17 Department of Breast Medical Oncology, The Cancer Institute Hospital of the Japanese Foundation for Cancer Research, 3-8-31 Ariake, Koto-ku, Tokyo, 135-8550 Japan; 18 Department of Breast Surgical Oncology, St. Luke’s International Hospital, 9-1 Akashi-cho, Chuo-ku, Tokyo, 104-8560 Japan; 19 Department of Breast and Endocrine Surgical Oncology, Tohoku University Graduate School of Medicine, Sendai, Miyagi 980-8575 Japan; 20 Department of Surgery and Science, Kyushu University, 3-1-1 Maidashi, Higashi-ku, Fukuoka, 812-8582 Japan; 21 Department of Breast and Endocrine Surgery, Kanagawa Cancer Center, 2-3-2 Nakao, Ashahi-ku, Yokohama, 241-8515 Japan; 22 Department of Medical Oncology, Fukushima Medical University, 1 Hikarigaoka, Fukushima, 960-1295 Japan; 23 The Japanese Breast Cancer Society Clinical Practice Guidelines for Systemic Treatment of Breast Cancer Panel Membership, Tokyo, Japan

**Keywords:** Breast cancer, Guideline, Systemic treatment

## Abstract

**Purpose:**

We present the English version of The Japanese Breast Cancer Society (JBCS) Clinical Practice Guidelines for systemic treatment of breast cancer, 2018 edition.

**Methods:**

The JBCS formed a task force to update the JBCS Clinical Practice Guidelines, 2015 edition, according to Minds Handbook for Clinical Practice Guideline Development 2014. First, we set multiple outcomes for each clinical question (CQ). Next, quantitative or qualitative systematic review was conducted for each of the multiple outcomes, and the strength of recommendation for the CQ was taken into consideration during meetings, with the aim of finding a balance between benefit and harm. Finalized recommendations from each session were confirmed through discussion and voting at the recommendation decision meeting.

**Results:**

The recommendations, the strength of recommendation and the strength of evidence were determined based on systemic literature reviews and the meta-analyses for each CQ.

**Conclusion:**

The JBCS updated the Clinical Practice Guidelines for systemic treatment of breast cancer.

**Electronic supplementary material:**

The online version of this article (10.1007/s12282-020-01085-0) contains supplementary material, which is available to authorized users.

## Introduction

Here we present the English version of “The Japanese Breast Cancer Society Clinical Practice Guidelines for systemic treatment of breast cancer, 2018 edition,” which was revised in May 2018, according to Minds Handbook for Clinical Practice Guidelines Development 2014 [1]. The latest version of the guidelines for systemic treatment of breast cancer, 2015 edition, was released but was not based on the Minds Handbook [2].

We based our recommendation, strength of recommendation (SoR) and strength of evidence (SoE) on a systematic quantitative and qualitative review according to Minds Handbook for Clinical Practice Guidelines Development 2014 [1, 3]. For each background question (BQ), we have supplied a statement, and for each clinical question (CQ), we have supplied a recommendation, SoR and SoE. Short explanations were added to CQ9 (adjuvant capecitabine combination therapy); CQ14 (second-line endocrine therapy (ET) for hormone receptor [HR]-positive/human epidermal growth factor receptor 2 (HER2)-negative metastatic breast cancer in pre- or perimenopausal patients); CQ15 (first-line ET for HR-positive/HER2-negative metastatic breast cancer in postmenopausal patients); CQ18 (first-line chemotherapy for HER2-negative metastatic breast cancer); and CQ22 (first-line therapy for HER2-positive metastatic breast cancer).

## Background questions

BQ1. Is adjuvant ET effective for HR-positive breast cancer?

Statement

Among patients with HR-positive breast cancer, strong evidence supports using tamoxifen in premenopausal patients and aromatase inhibitors (AIs) in postmenopausal patients.

BQ2. Should the type of adjuvant ET be considered in determining the optimal first-line ET regimen for HR-positive metastatic breast cancer?

Statement

The type of adjuvant ET, and disease-free interval, should be considered in determining the optimal first-line ET regimen for HR-positive metastatic breast cancer.

BQ3. What is the best method for suppressing/ablating ovarian function with metastatic or recurrent HR-positive breast cancer in premenopausal patients?

Statement

Three methods are used for suppressing/ablating ovarian function: use of luteinizing hormonereleasing hormone (LH–RH) agonists, bilateral oophorectomy, and irradiation. However, the optimal method for ovarian function suppression is unclear. LH–RH agonists are generally used in clinical practice, but toxicity, cost, and treatment duration should be considered in selecting this method.

BQ4. Does response to prior ET predict the efficacy of subsequent ET for HR-positive metastatic breast cancer?

Statement

Generally, patients who respond well to prior ET tend to respond to subsequent ET. However, even some patients who do not respond well to prior ET have good responses to subsequent ET.

BQ5. Are bone-modifying agents (bisphosphonates, denosumab) recommended for breast cancer patients with bone metastases?

Statement

1. Bone-modifying agents, such as zoledronic acid and denosumab, reduce the risk of skeletal-related events (SRE) and are a standard of care for patients with bone metastases.
2. Although the efficacy of zoledronic acid administered over a 12-week course is non-inferior to a 3- to 4-week course for selected breast cancer patients with bone metastases, long-term efficacy and safety are unclear.
3. As denosumab has been shown to reduce SRE risk compared with zoledronic acid, denosumab might be favored over zoledronic acid.
4. Osteonecrosis of the jaw (ONJ) due to bone-modifying agents lowers the patient's quality of life. Therefore, ONJ risk should be evaluated before starting bone-modifying agents, and patients should receive regular oral care during this treatment.

BQ6. Is ET or chemotherapy recommended as initial treatment for metastatic HR-positive/HER2-negative breast cancer?

Statement

Among patients with metastatic HR-positive/HER2-negative breast cancer, ET is usually recommended as initial treatment for breast cancer that is not rapidly progressing and has no visceral metastases, and chemotherapy is usually recommended as initial treatment for breast cancer that shows rapid progression, visceral metastasis, and/or de novo ET resistance.

BQ7. Is intra-arterial infusion chemotherapy a treatment option for metastatic breast cancer?

Statement

Intra-arterial infusion chemotherapy should not be performed as a routine clinical practice.

BQ8. Are the recommendations for adjuvant systemic therapy for special types of breast cancer the same as those for invasive ductal carcinoma (IDC)?

Statement

Systemic therapy should be considered for special types of breast cancer according to their histological classification. ET alone is recommended for HR-positive, lymph node-negative, pure mucinous breast carcinoma. ET alone or no systemic therapy is recommended for HR-positive, lymph node-negative tubular carcinoma.

Chemotherapy can be omitted for lymph node-negative adenoid cystic carcinoma, even if it has a triple negative phenotype. Systemic therapies for medullary carcinoma, apocrine carcinoma, and invasive lobular carcinoma should be considered in conformity with the recommendation for IDC.

BQ9. How should treatment strategy be determined for women with adenocarcinoma in axillary lymph nodes, but with an unknown primary site?

Statement

Breast cancer should be suspected in women with adenocarcinoma in axillary lymph nodes from unknown primary sites. Biopsy materials from these patients should be immunohistochemically tested for estrogen receptor, progesterone receptor, and HER2 before treatment, both to support a diagnosis of breast cancer and to plan treatment strategies for these patients. Treatment strategy should be determined according to the recommendation of systemic therapy for patients with node-positive breast cancer.

BQ10. Are multidisciplinary treatments recommended for locally advanced breast cancer or inflammatory breast cancer?

Statement

For locally advanced or inflammatory breast cancer, multidisciplinary treatment, such as chemotherapy followed by appropriate local treatments (surgery, radiotherapy), is considered standard of care.

BQ11. Is influenza vaccination or pneumococcal vaccination recommended before chemotherapy?

Statement

1. Vaccination for influenza is recommended prior to chemotherapy for breast cancer.
2. Less information is available regarding the usefulness of pneumococcal vaccination; however, inoculation may be prudent prior to chemotherapy for breast cancer.

BQ12. Is medication required for hot flashes or joint pain due to ET?

Statement

1. Hormone replacement therapy should not be used to treat hot flashes caused by ET.
2. Although selective serotonin reuptake inhibitors (SSRIs) help reduce hot flashes, whether their use increases risk of breast cancer recurrence in women taking SSRIs with tamoxifen is unclear.
3. For joint pain, drug therapy, such as nonsteroidal anti-inflammatory drugs or acetaminophen, is recommended.

BQ13. Are complementary and alternative therapies recommended for breast cancer treatment?

Statement

1. Complementary or alternative therapies should not be given with the aim of suppressing breast cancer progression or prolonging survival.
2. Complementary or alternative therapies may be considered for the purpose of alleviating symptoms associated with standard cancer treatment or relieving anxiety.

BQ14. Are bone-modifying agents (bisphosphonate, denosumab) recommended to prevent and treat osteoporosis in patients treated with AI?

Statement

Among patients treated with AIs, periodic bone density measurements and use of bone-modifying agents according to risk for fracture are the standard of care.

## Clinical questions

CQ1. What adjuvant ET is recommended for HR-positive breast cancer in pre- or perimenopausal patients?

Recommendations

1. Tamoxifen is strongly recommended (SoR: 1, SoE: strong).
2. Combination use of an LH–RH agonist and tamoxifen is weakly recommended (SoR: 2, SoE: strong).
3. Combination use of an LH–RH agonist and an AI is weakly recommended (SoR: 2, SoE: moderate).

CQ2. What is recommended as adjuvant ET for HR-positive breast cancer in postmenopausal patients?

Recommendations

1. AI is strongly recommended (SoR: 1, SoE: strong).
2. Tamoxifen for 2–3 years followed by AI for a total of 5 years is weakly recommended (SoR: 2, SoE: strong).
3. AI for 2 years followed by tamoxifen for a total of 5 years is weakly recommended (SoR: 2, SoE: weak).
4. Tamoxifen or toremifene is weakly recommended (SoR: 2, SoE: strong).

CQ3. What is the optimal duration of adjuvant ET for invasive HR-positive breast cancer?

Recommendations

1. For pre- or perimenopausal women who are diagnosed with HR-positive breast cancer, tamoxifen is strongly recommended for a total duration of 10 years (SoR: 1, SoE: moderate).
2. For postmenopausal women who are diagnosed with HR-positive breast cancer, AI is weakly recommended for a total duration of 10 years (SoR: 2, SoE: moderate).

CQ4. Is neoadjuvant ET recommended for invasive HR-positive breast cancer?

CQ4-a. For postmenopausal women

Recommendations

Among postmenopausal women with operable HR-positive breast cancers, the rate of breast-conserving therapy for those who receive neoadjuvant ET reported equal to that for women who receive chemotherapy. However, optimal treatment duration and neoadjuvant ET regimen are unclear. Therefore, we cannot state a recommendation at this time (SoR: 2–3, SoE: weak).

CQ4-b. For pre- or perimenopausal women

Recommendation

For pre- or perimenopausal women with operable invasive HR-positive breast cancer, neoadjuvant ET is not recommended because the rate of breast-conserving therapy is reportedly inferior to that in women who receive neoadjuvant chemotherapy (SoR: 3, SoE: very weak).

CQ5. Is adjuvant ET recommended for HR-positive ductal carcinoma in situ (DCIS)?

Recommendations

Tamoxifen is weakly recommended for pre- or perimenopausal patients with HR-positive DCIS who receive breast-conserving therapy to reduce the risk of ipsilateral breast cancer. For postmenopausal patients who receive breast-conserving therapy, tamoxifen or AI is weakly recommended (SoR: 2, SoE: moderate).

CQ6. Is neoadjuvant chemotherapy recommended for operable invasive breast cancer?

Recommendation

For operable invasive breast cancer, neoadjuvant chemotherapy is weakly recommended to facilitate breast-conserving surgery (SoR: 2, SoE: strong).

CQ7. Is neoadjuvant chemotherapy combined with trastuzumab recommended for invasive, operable HER2-positive breast cancer?

Recommendation

When neoadjuvant chemotherapy is planned for invasive, operable HER2-positive breast cancer, adding trastuzumab to chemotherapy is strongly recommended, because chemotherapy plus trastuzumab is shown to increase pathological complete response rate compared with chemotherapy alone for invasive, operable HER2-positive breast cancer (SoR: 1, SoE: moderate).

CQ8. Is oral fluoride pyrimidine recommended instead of conventional intravenous chemotherapy as adjuvant chemotherapy in breast cancer?

Recommendation

Oral fluoride pyrimidine is not recommended as adjuvant chemotherapy instead of conventional intravenous chemotherapy for breast cancer patients (SoR: 3, SoE: weak).

CQ9. Is concurrent use of capecitabine in conventional intravenous chemotherapy recommended as adjuvant chemotherapy for breast cancer?

Recommendations

Because adding capecitabine to conventional chemotherapy may be effective as adjuvant chemotherapy, this combination therapy is weakly recommended (SoR: 2, SoE: moderate).

Two randomized phase III trials that examined the addition of capecitabine to conventional adjuvant chemotherapy, including anthracycline- and taxane-based regimens, have been reported [4, 5]. Although the results of these trials showed that disease-free survival (DFS), which was the primary end point of these trials, was not significantly improved by adding capecitabine to conventional adjuvant chemotherapy, overall survival (OS) was shown to improve in one of these trials [4]. We conducted an integrated analysis of these trials in this guideline and found that adding capecitabine to conventional adjuvant chemotherapy significantly improved OS (hazard ratio 0.77, 95% confidence interval [CI] 0.64–0.93), but not DFS (hazard ratio 0.86, 95% CI 0.74–1.01; Supplemental Fig. 1). We consider that adding capecitabine to conventional adjuvant chemotherapy has modest efficacy. Adjuvant capecitabine therapy is not approved and not reimbursed in Japan. Therefore, adding capecitabine to conventional chemotherapy is weakly recommended as an adjuvant chemotherapy.

CQ10. Is adjuvant chemotherapy combined with trastuzumab recommended for HER2-positive breast cancer?

Recommendation

Chemotherapy combined with trastuzumab is strongly recommended as adjuvant therapy for HER2-positive breast cancer (SoR: 1, SoE: strong).

CQ11. Is dose-dense chemotherapy recommended as adjuvant therapy for breast cancer patients with high recurrence risk and adequate bone marrow function?

Recommendation

Dose-dense chemotherapy with granulocyte-colony stimulating factor support is strongly recommended as adjuvant chemotherapy for patients who have high recurrence risk and sufficient bone marrow function (SoR: 1, SoE: strong).

CQ12. Should Ki67 be considered as an indication for adjuvant chemotherapy for HR-positive/HER2-negative breast cancer?

Recommendation

Although Ki67 is a prognostic marker, some methodological issues regarding Ki67 evaluation (e.g., inter-institutional discordance) have been reported. However, high Ki67 status might be considered an indication for adjuvant chemotherapy in HR-positive/HER2-negative node-negative breast cancer (SoR: 2, SoE: weak).

CQ13. What is recommended as first-line ET for metastatic HR-positive/HER2-negative breast cancer in pre- or perimenopausal patients?

Recommendations

1. A combination of an ovarian function suppressor, such as LH–RH agonist or bilateral oophorectomy, and tamoxifen is strongly recommended (SoR: 1, SoE: strong).
2. A combination of an ovarian function suppressor, such as LH–RH agonist or bilateral oophorectomy, and ET recommended for postmenopausal patients as first-line treatment is weakly recommended (SoR: 2, SoE: weak).

CQ14. What is recommended as second-line ET for metastatic HR-positive/HER2-negative breast cancer in pre- or perimenopausal patients?

Recommendations

1. A combination therapy of LH-RH agonist plus fulvestrant plus cyclin-dependent kinase (CDK) 4/6 inhibitor is weakly recommended (SoR: 2, SoE: moderate).
2. A combination of an ovarian function suppressor and ET recommended for postmenopausal patients is weakly recommended (SoR: 2, SoE: moderate).

Although few data are available about the optimal sequencing of ET for metastatic HR-positive/HER2-negative breast cancer in pre- and perimenopausal patients, second-line or later ET is often based on the type and efficacy of previous endocrine therapies (please refer to BQ2).

The PALOMA-3 trial was a randomized controlled trial that included post-, peri- and premenopausal patients with metastatic breast cancer that had relapsed or progressed during prior ET; it showed the efficacy of the combination of palbociclib (a CDK4/6 inhibitor), fulvestrant, and goserelin [6]. About 21% of the PALOMA-3 trial’s cohort were pre- or perimenopausal patients. Subgroup analysis of these patients showed that median progression-free survival (PFS) in the palbociclib arm was 9.5 months versus 5.6 months for the placebo arm (hazard ratio 0.50, 95% CI 0.29–0.87) [7].

CQ15. What is recommended as first-line ET for HR-positive/HER2-negative metastatic breast cancer in postmenopausal patients?

Recommendations

1. AI is strongly recommended (SoR: 1, SoE: strong).
2. A combination of AI and CDK4/6 inhibitor is strongly recommended (SoR: 1, SoE: strong).
3. Fulvestrant (500 mg) is strongly recommended (SoR: 1, SoE: strong).

Several randomized controlled trials of first-line ET for HR-positive metastatic breast cancer in postmenopausal patients have compared AI monotherapy with other ETs, including tamoxifen. Some meta-analyses reported that AI monotherapy significantly prolonged OS compared with tamoxifen or other ETs [8, 9]. However, another meta-analysis of the third-generation AI compared with tamoxifen showed no statistical difference between them regarding OS, although the overall response rate (ORR) and clinical benefit rate (CBR) of AI were better than those of tamoxifen (odds ratio [OR] 1.56, 95% CI 1.17–2.07 and OR 1.70, 95% CI 1.24–2.33, respectively) [10].

We performed a meta-analysis of four randomized controlled trials—the PALOMA-1, PALOMA-2, MONALEESA-2, and MONARCH 3 trials—which compared the combination of AI and CDK4/6 inhibitor to AI monotherapy in first-line settings [11–14]. This meta-analysis demonstrated that PFS (relative risk [RR] 0.67, 95% CI 0.60–0.73), ORR (risk difference [RD] 0.11, 95% CI 0.07–0.16) and CBR (RD 0.11, 95% CI 0.07–0.15) were consistently better in the combined AI and CDK4/6 inhibitor arm (Supplemental Fig. 2). However, this combination arm also had a higher rate of adverse events of grade 3 or above (RD 0.43, 95% CI 0.39–0.47).

We performed integrated analysis of two randomized controlled trials comparing fulvestrant (500 mg) with anastrozole as first-line ET for HR-positive metastatic breast cancer in postmenopausal patients. One trial is the randomized phase II FIRST trial and the other trial is the randomized phase III FALCON trial [15–17]. We found that PFS and time to progression (TTP) were better with fulvestrant than with anastrozole (Supplemental Fig. 3).

CQ16. What is recommended as second-line ET for metastatic HR-positive/HER2-negative breast cancer in postmenopausal patients?

CQ16-a. AI-resistant breast cancer

Recommendations

1. A combination of fulvestrant and CDK4/6 inhibitor is strongly recommended (SoR: 1, SoE: strong).
2. Fulvestrant (500 mg) is strongly recommended (SoR: 1, SoE: strong).
3. Everolimus in combination with exemestane is weakly recommended for non-steroidal, AI-resistant breast cancer (SoR: 2, SoE: strong).
4. Exemestane is weakly recommended for non-steroidal, AI-resistant breast cancer (SoR: 2, SoE: moderate).
5. Tamoxifen or toremifene is weakly recommended (SoR: 2, SoE: weak).

CQ16-b. Tamoxifen-resistant breast cancer

Recommendations

AI is strongly recommended for tamoxifen-resistant breast cancer (SoR: 1, SoE: strong).

CQ17. What is recommended as third-line and later ET for metastatic HR-positive/HER2-negative breast cancer in postmenopausal patients?

Recommendations

1. A combination of fulvestrant and CDK4/6 inhibitor is weakly recommended (SoR: 2, SoE: moderate).
2. Everolimus combined with exemestane is weakly recommended for exemestane-naïve and non-steroidal, AI-resistant disease (SoR: 2, SoE: moderate).
3. ETs not used in first- and second lines are weakly recommended (SoR: 2, SoE: weak).

CQ18. What is recommended as first-line chemotherapy for previously untreated metastatic HER2-negative cancer in patients who did not receive anthracycline and taxanes as neoadjuvant or adjuvant therapy?

Recommendations

1. Anthracycline or taxanes are strongly recommended (SoR: 1, SoE: moderate).
2. S-1 is weakly recommended (SoR: 2, SoE: moderate).
3. Treatment with capecitabine (SoR: 3, SoE: moderate), gemcitabine (SoR: 3, SoE: moderate), vinorelbine (SoR: 3, SoE: moderate), or eribulin (SoR: 3, SoE: moderate) is not recommended.
  1. *Anthracycline* We conducted a meta-analysis of eight trials that compared anthracycline-containing regimens with regimens that did not include both anthracyclines and taxanes, such as CMF (cyclophosphamide, methotrexate, fluorouracil), for first-line chemotherapy of metastatic breast cancer. Anthracycline-containing regimens were significantly superior to regimens that did not include both anthracyclines and taxanes for OS (hazard ratio 0.79, 95% CI 0.69–0.92) and ORR (hazard ratio 1.29, 95% CI 1.07–1.56; Supplemental Fig. 4).
  2. *Taxane* We conducted a meta-analysis of five trials to compare taxane monotherapy regimens with anthracycline-containing regimens for the first-line chemotherapy of metastatic breast cancer that showed that OS, PFS, and ORR were equivalent in both regimens (Supplemental Fig. 5). Two trials compared quality of life (QOL) in anthracycline-containing regimens with that in taxane-containing regimens and found no difference in QOL between the two therapies [18, 19].
  3. *S-1* S-1 (40–60 mg twice daily for 28 consecutive days, followed by a 14-day break) is an oral fluorouracil derivative used in Japan. The SELECT BC trial showed that S-1 was not inferior to taxanes in OS (hazard ratio 1.05, 95% CI 0.86–1.27), although S-1 was not shown to be non-inferior to taxanes in PFS (hazard ratio 1.18, 95% CI 0.99–1.40, which exceeded the non-inferiority margin of 1.33) [20]. QOL evaluation in the SELECT BC trial showed that S-1 was superior to taxanes.
  4. *Capecitabine* One trial compared capecitabine with pegylated liposomal doxorubicin hydrochloride as first-line treatment among patients aged 65 years and older [21]. This study reported no difference in OS and PFS between these groups. However, this study was considered to have critical limitations, as the cohort was very small (*n* = 78) and included only elderly patients.
  5. *Gemcitabine* One trial compared gemcitabine monotherapy with epirubicin monotherapy in first-line setting among patients aged 60 years or older [22]. The results of this study showed that the epirubicin group was superior to the gemcitabine group in OS, TTP, and ORR.
  6. *Vinorelbine* To our knowledge, no trials have compared vinorelbine monotherapy with anthracyclines or taxanes as first-line treatments.
  7. *Eribulin* To our knowledge, no randomized trials have compared eribulin with anthracyclines or taxanes as first-line treatments.

CQ19. What is recommended for second-line or later chemotherapy for HER2-negative metastatic breast cancer in patients who did not receive anthracycline and taxanes as neoadjuvant or adjuvant therapy?

Recommendations

1. Anthracycline, taxanes or S-1 (whichever were not used as the first-line chemotherapy) are strongly recommended. Capecitabine or eribulin is also strongly recommended (SoR: 1, SoE: moderate).
2. Gemcitabine or vinorelbine is weakly recommended (SoR: 2, SoE: moderate).

CQ20. Is combination therapy with bevacizumab recommended as first- or second-line treatment for HER2-negative metastatic breast cancer?

Recommendation

Chemotherapy in combination with bevacizumab as first- or second-line for metastatic HER2-negative breast cancer is weakly recommended (SoR: 2, SoE: moderate).

CQ21. Is combination chemotherapy recommended for HER2-negative metastatic breast cancer?

Recommendation

In general, combination chemotherapy, such as doxorubicin plus paclitaxel, is not recommended for metastatic breast cancer (SoR: 3, SoE: weak).

CQ22. What is recommended as first-line therapy for HER2-positive metastatic breast cancer?

Recommendations

1. The combination of trastuzumab, pertuzumab and docetaxel is strongly recommended (SoR: 1, SoE: strong).
2. The combination of trastuzumab and chemotherapy is weakly recommended (SoR: 2, SoE: moderate).
3. Trastuzumab emtansine (T-DM1) is weakly recommended (SoR: 2, SoE: moderate).
  1. The CLEOPATRA trial showed that adding pertuzumab to trastuzumab and docetaxel improved PFS (hazard ratio 0.66, 95% CI 0.58–0.81) [23] and OS (hazard ratio 0.68, 95% CI 0.56–0.84) [24] compared with placebo plus trastuzumab plus docetaxel. Although adding pertuzumab increased the incidences of neutropenia, febrile neutropenia, and diarrhea of grade 3 or above [23], the benefit of the combination therapy with pertuzumab is considered to exceed its harm. This study was well designed and SoE was estimated as strong. Therefore, the combination of trastuzumab, pertuzumab and docetaxel is strongly recommended.
  2. Some studies evaluated the combination of trastuzumab and chemotherapy [25–27]. The results of the meta-analysis we performed showed that combining taxane and trastuzumab improved PFS (hazard ratio 0.53, 95% CI 0.42–0.68) and OS (hazard ratio 0.80, 95% CI 0.65–0.99) compared with taxane alone as first-line therapy for HER2-positive metastatic breast cancer (Supplemental Fig. 6). Two studies reported that combining vinorelbine and trastuzumab showed similar efficacy to combining taxane and trastuzumab [28, 29]. As described above, the CLEOPATRA study found that the efficacy of combining docetaxel and trastuzumab without pertuzumab was inferior to that of the combination therapy with pertuzumab; therefore, combining trastuzumab and chemotherapy is weakly recommended.
  3. The result of the MARIANNE trial revealed that the efficacy of T-DM1 was non-inferior, but not superior, to that of combining taxane and trastuzumab [30]. The incidence of adverse events of grade 3 or above was lower in T-DM1 than the combination of taxane and trastuzumab. As first-line treatment for HER2-positive metastatic breast cancer, T-DM1 was not shown to prolong PFS compared with the combination of trastuzumab, pertuzumab and docetaxel, although there are no trials of their direct comparison, but was less toxic than taxane plus trastuzumab. Therefore, T-DM1 could be considered as an option for first-line treatment of HER2-positive metastatic breast cancer.

CQ23. What is recommended as second-line therapy for HER2-positive metastatic breast cancer?

Recommendations

1. T-DM1 is strongly recommended for HER2-positive metastatic breast cancer in patients who experience disease progression after receiving a trastuzumab-containing regimen (SoR: 1, SoE: moderate).
2. The combination of chemotherapy and trastuzumab is weakly recommended (SoR: 2, SoE: weak).
3. Combination therapy of capecitabine and lapatinib is not recommended (SoR: 3, SoE: moderate).

CQ24. What is recommended as third-line therapy for HER2-positive metastatic breast cancer?

Recommendations

1. T-DM1 is weakly recommended for HER2-positive metastatic breast cancer if not previously administered (SoR: 2, SoE: moderate).
2. Anti-HER2 agents plus an alternative cytotoxic agent may be recommended as third-line or later therapy for patients with HER2-positive metastatic breast cancer who had experienced disease progression on a HER2-directed agent-containing regimen (SoR: 2, SoE: weak).

CQ25. Is ET alone or ET in combination with anti-HER2 therapy recommended for patients with HR-positive/HER2-positive metastatic breast cancer for whom chemotherapy is contraindicated?

Recommendations

1. ET combined with anti-HER2 therapy is weakly recommended (SoR: 2, SoE: moderate).
2. ET used alone is not recommended (SoR: 3, SoE: moderate).

CQ26. What is recommended as adjuvant therapy for breast cancer in elderly patients?

CQ26-a. Adjuvant ET

Recommendation

AI or tamoxifen is strongly recommended as adjuvant ET for elderly patients with HR-positive breast cancer (SoR: 1, SoE: weak).

CQ26-b. Adjuvant chemotherapy

Recommendation

Standard chemotherapy may be recommended as adjuvant chemotherapy for elderly patients with breast cancer, although treatment should be individualized based on prognosis, comprehensive geriatric assessment (e.g., VES-13), and patient preference (SoR: 1–2, SoE: strong).

CQ26-c. Adjuvant anti-HER2 therapy in combination with chemotherapy

Recommendation

Chemotherapy combined with anti-HER2 therapy is strongly recommended as an adjuvant therapy for elderly patients with HER2-positive breast cancer, although treatment should be individualized based on prognosis, global health status, and patient preference (SoR: 1, SoE: weak).

CQ27. What systemic therapy is recommended for metastatic breast cancer in elderly patients?

Recommendations

1. ET is strongly recommended for elderly patients with metastatic HR-positive breast cancer, although treatment consideration should be individualized based on prognosis, global health status, and patient preference (SoR: 1, SoE: very weak).
2. Chemotherapy may be needed for elderly patients with metastatic breast cancer, although treatment consideration should be individualized based on prognosis, global health status, and patient preference (SoR: 2, SoE: very weak).
3. Combination treatment with molecular targeted therapy may be needed for elderly patients with metastatic breast cancer, although treatment should be individualized, based on prognosis, global health status, and patient preference (SoR: 2, SoE: very weak).

## Conclusion

The JBCS updated the Clinical Practice Guidelines for systemic treatment of breast cancer, according to Minds Handbook for Clinical Practice Guideline Development 2014.

## Electronic supplementary material

Below is the link to the electronic supplementary material.

Supplemental Figure 1. Integrated analysis of conventional chemotherapy, with or without concurrent adjuvant use of capecitabine, for patients with breast cancer. (a) Overall survival, (b) disease-free survival. (PPTX 52 kb)

Supplemental Figure 2. Meta-analysis of aromatase inhibitor, with or without concurrent use of cyclin-dependent kinase 4/6 inhibitor, as first-line therapy for postmenopausal patients with metastatic breast cancer. (a) Progression-free survival, (b) overall response rate, (c) clinical benefit rate. (PPTX 64 kb)

Supplemental Figure 3. Integrated analysis comparing fulvestrant to anastrozole as first-line endocrine therapy for metastatic breast cancer in postmenopausal patients. (PPTX 41 kb)

Supplemental Figure 4. Meta-analysis comparing anthracycline-containing regimens with regimens that use both anthracyclines and taxanes (such as CMF), as first-line chemotherapy for patients with metastatic breast cancer. (a) Overall survival, (b) overall response rate. (PPTX 123 kb)

Supplemental Figure 5. Meta-analysis comparing taxane monotherapy with anthracycline-containing regimens as first-line chemotherapy for patients with metastatic breast cancer. (a) Overall survival, (b) progression-free survival. (PPTX 133 kb)

Supplemental Figure 5. Meta-analysis comparing taxane monotherapy with anthracycline-containing regimens as first-line chemotherapy for patients with metastatic breast cancer (c) overall response rate (PPTX 81 kb)

Supplemental Figure 6. Meta-analysis comparing taxane + trastuzumab with taxane-only as first-line therapy for patients with HER2-positive metastatic breast cancer. (a) Overall survival, (b) progression-free survival. (PPTX 266 kb)

## Abbreviations

AI: Aromatase inhibitor
BQ: Background question
CBR: Clinical benefit rate
CDK: Cyclin-dependent kinase
CI: Confidence interval
CQ: Clinical question
DCIS: Ductal carcinoma in situ
DFS: Disease-free survival
ET: Endocrine therapy
HER2: Human epidermal growth factor receptor-2
HR: Hormone receptor
IDC: Invasive ductal carcinoma
LH-RH: Luteinizing hormone-releasing hormone
ONJ: Osteonecrosis of the jaw
OR: Odds ratio
ORR: Overall response rate
OS: Overall survival
PFS: Progression-free survival
QOL: Quality of life
RD: Risk difference
RR: Relative risk
SoE: Strength of evidence
SoR: Strength of recommendation
SRE: Skeletal-related events
SSRIs: Selective serotonin reuptake inhibitors
T-DM1: Trastuzumab emtansine
TTP: Time to progression
