# Correction to: The Japanese Breast Cancer Society Clinical Practice Guidelines for systemic treatment of breast cancer, 2018 edition

**DOI:** 10.1007/s12282-021-01252-x

**Published:** 2021-04-22

**Authors:** Tatsunori Shimoi, Shigenori E. Nagai, Tetsuhiro Yoshinami, Masato Takahashi, Hitoshi Arioka, Mikiya Ishihara, Yuichiro Kikawa, Kei Koizumi, Naoto Kondo, Yasuaki Sagara, Masahiro Takada, Toshimi Takano, Junji Tsurutani, Yoichi Naito, Rikiya Nakamura, Masaya Hattori, Fimikata Hara, Naoki Hayashi, Toshiro Mizuno, Minoru Miyashita, Nami Yamashita, Takashi Yamanaka, Shigehira Saji, Hiroji Iwata, Tatsuya Toyama

**Affiliations:** 1 Department of Breast and Medical Oncology, National Cancer Center Hospital, 5-1-1 Tsukiji,, Chuo-ku, Tokyo 104-0045 Japan; 2 Department of Breast Oncology, Saitama Cancer Center, 780 Komuro, Ina-machi, Kitaadachi-gun, Saitama, 362-0806 Japan; 3 Department of Breast and Endocrine Surgery, Graduate School of Medicine, Osaka University, 2-2-E 10 Yamadaoka, Suita, Osaka 565-0871 Japan; 4 Department of Breast Surgery, NHO Hokkaido Cancer Center, 4-2 Kikusui, Shiroishi-ku, Sapporo, 003-0804 Japan; 5 Department of Medical Oncology, Yokohama Rosai Hospital, 3211 Kozukue, Kohoku-ku, Yokohama, Kanagawa 222-0036 Japan; 6 Department of Medical Oncology, Mie University Hospital, 2-174 Edobashi, Tsu, Mie 514-8507 Japan; 7 Department of Breast Surgery, Kobe City Medical Center General Hospital, 2-1-1, Minatojimaminamimachi, Chuo-ku, Kobe, Hyogo 650-0047 Japan; 8 First Department of Surgery, Hamamatsu University School of Medicine, 1-20-1 Handayama, Higashi-ku, Hamamatsu City, Shizuoka 431-3192 Japan; 9 Department of Breast Surgery, Nagoya City University Graduate School of Medical Sciences, 1 Kawasumi, Mizuho-cho, Mizuho-ku, Nagoya 467-8601 Japan; 10 Department of Breast Surgical Oncology, Hakuaikai Social Cooperation, Sagara Hospital, 3-31 Matsubara-cho, Kagoshima, 892-0098 Japan; 11 Department of Breast Surgery, Kyoto University Hospital, 54 Kawaharacho, Shogoin, Sakyo-ku, Kyoto, 606-8507 Japan; 12 Department of Medical Oncology, Toranomon Hospital, 2-2-2 Toranomon, Minato-ku, Tokyo, 105-8470 Japan; 13 Department of Medical Oncology, Advanced Cancer Translational Research Institute, Showa University, 1-5-8 Hatanodai, Shinagawa, Tokyo Japan; 14 Department of Breast and Medical Oncology, National Cancer Center Hospital East, 6-5-1 Kashiwanoha, Kashiwa, Chiba 277-8577 Japan; 15 Department of Breast Surgery, Chiba Cancer Center, 666-2 Nitona-cho, Chuo-ku, Chiba, Chiba 280-8717 Japan; 16 Department of Breast Oncology, Aichi Cancer Center, 1-1 Kanokoden, Chikusa-ku, Nagoya, 464-8681 Japan; 17 Department of Breast Medical Oncology, The Cancer Institute Hospital of the Japanese Foundation for Cancer Research, 3-8-31 Ariake, Koto-ku, Tokyo, 135-8550 Japan; 18 Department of Breast Surgical Oncology, St. Luke’s International Hospital, 9-1 Akashi-cho, Chuo-ku, Tokyo, 104-8560 Japan; 19 Department of Breast and Endocrine Surgical Oncology, Tohoku University Graduate School of Medicine, Sendai, Miyagi 980-8575 Japan; 20 Department of Surgery and Science, Kyushu University, 3-1-1 Maidashi, Higashi-ku, Fukuoka, 812-8582 Japan; 21 Department of Breast and Endocrine Surgery, Kanagawa Cancer Center, 2-3-2 Nakao, Ashahi-ku, Yokohama, 241-8515 Japan; 22 Department of Medical Oncology, Fukushima Medical University, 1 Hikarigaoka, Fukushima, 960-1295 Japan; 23 The Japanese Breast Cancer Society Clinical Practice Guidelines for Systemic Treatment of Breast Cancer Panel Membership, Tokyo, Japan

## Correction to: Breast Cancer (2020) 27:322–331 10.1007/s12282-020-01085-0

The article “The Japanese Breast Cancer Society Clinical Practice Guidelines for systemic treatment of breast cancer, 2018 edition”, written by Tatsunori Shimoi, Shigenori E. Nagai, Tetsuhiro Yoshinami, Masato Takahashi, Hitoshi Arioka, Mikiya Ishihara, Yuichiro Kikawa, Kei Koizumi, Naoto Kondo, Yasuaki Sagara, Masahiro Takada, Toshimi Takano, Junji Tsurutani, Yoichi Naito, Rikiya Nakamura, Masaya Hattori, Fimikata Hara, Naoki Hayashi, Toshiro Mizuno, Minoru Miyashita, Nami Yamashita, Takashi Yamanaka, Shigehira Saji, Hiroji Iwata, Tatsuya Toyama, was originally published electronically on the publisher’s internet portal on 2 April 2020 without open access. After publication in volume 27, issue 3, page 322–331, the author decided to opt for Open Choice and to make the article an Open Access publication. Therefore, the copyright of the article has been changed to © The Author(s) and the article is forthwith distributed under the terms of the Creative Commons Attribution 4.0 International License, which permits use, sharing, adaptation, distribution and reproduction in any medium or format, as long as you give appropriate credit to the original author(s) and the source, provide a link to the Creative Commons licence, and indicate if changes were made.

The images or other third party material in this article are included in the article’s Creative Commons licence, unless indicated otherwise in a credit line to the material. If material is not included in the article’s Creative Commons licence and your intended use is not permitted by statutory regulation or exceeds the permitted use, you will need to obtain permission directly from the copyright holder.

To view a copy of this licence, visit http://creativecommons.org/licenses/by/4.0/.

The original article has been updated.

