# Supplementary material for: The Japanese Breast Cancer Society Clinical Practice Guidelines for systemic treatment of breast cancer, 2018 edition
Source: Breast Cancer. 2020 Apr 2;27(3):322–31. doi: 10.1007/s12282-020-01085-0 (PMC8062371; doi:10.1007/s12282-020-01085-0)
Supplement: Supplementary file 1 — Supplemental Figure 1. Integrated analysis of conventional chemotherapy, with or without concurrent adjuvant use of capecitabine, for patients with breast cancer. (a) Overall survival, (b) disease-free survival. (PPTX 52 kb) [file 12282_2020_1085_MOESM1_ESM.pptx]

## Slide 1
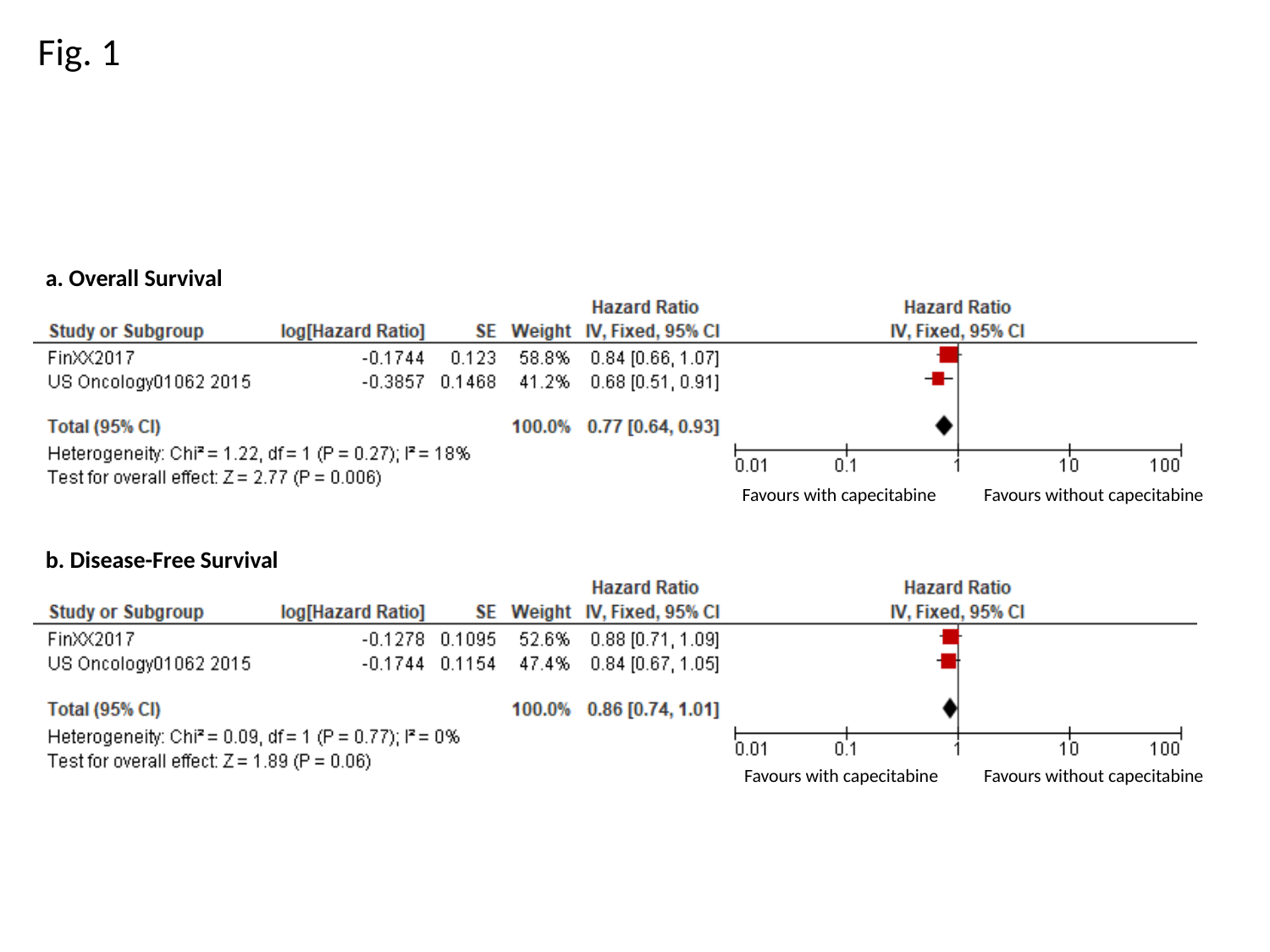

# Fig. 1
a. Overall Survival
Favours without capecitabine
Favours with capecitabine
b. Disease-Free Survival
Favours without capecitabine
Favours with capecitabine
