# Supplementary figures and images for: The Japanese Breast Cancer Society Clinical Practice Guidelines for systemic treatment of breast cancer, 2018 edition
Source: Breast Cancer. 2020 Apr 2;27(3):322–31. doi: 10.1007/s12282-020-01085-0 (PMC8062371; doi:10.1007/s12282-020-01085-0)

## Slide 1
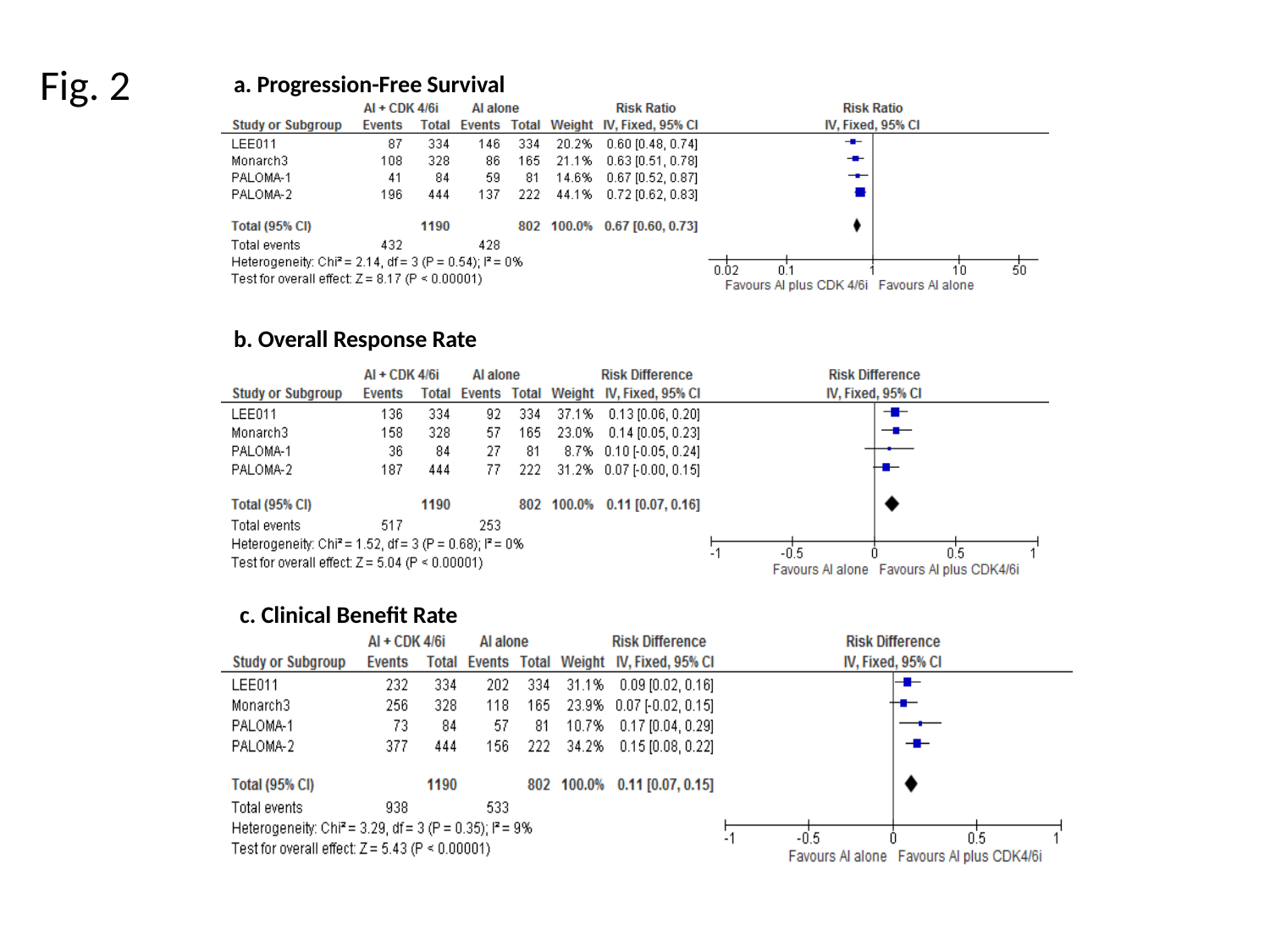

# Fig. 2
a. Progression-Free Survival
b. Overall Response Rate
c. Clinical Benefit Rate

Supplement: Supplementary file 2 — Supplemental Figure 2. Meta-analysis of aromatase inhibitor, with or without concurrent use of cyclin-dependent kinase 4/6 inhibitor, as first-line therapy for postmenopausal patients with metastatic breast cancer. (a) Progression-free survival, (b) overall response rate, (c) clinical benefit rate. (PPTX 64 kb) [file 12282_2020_1085_MOESM2_ESM.pptx]

## Slide 1
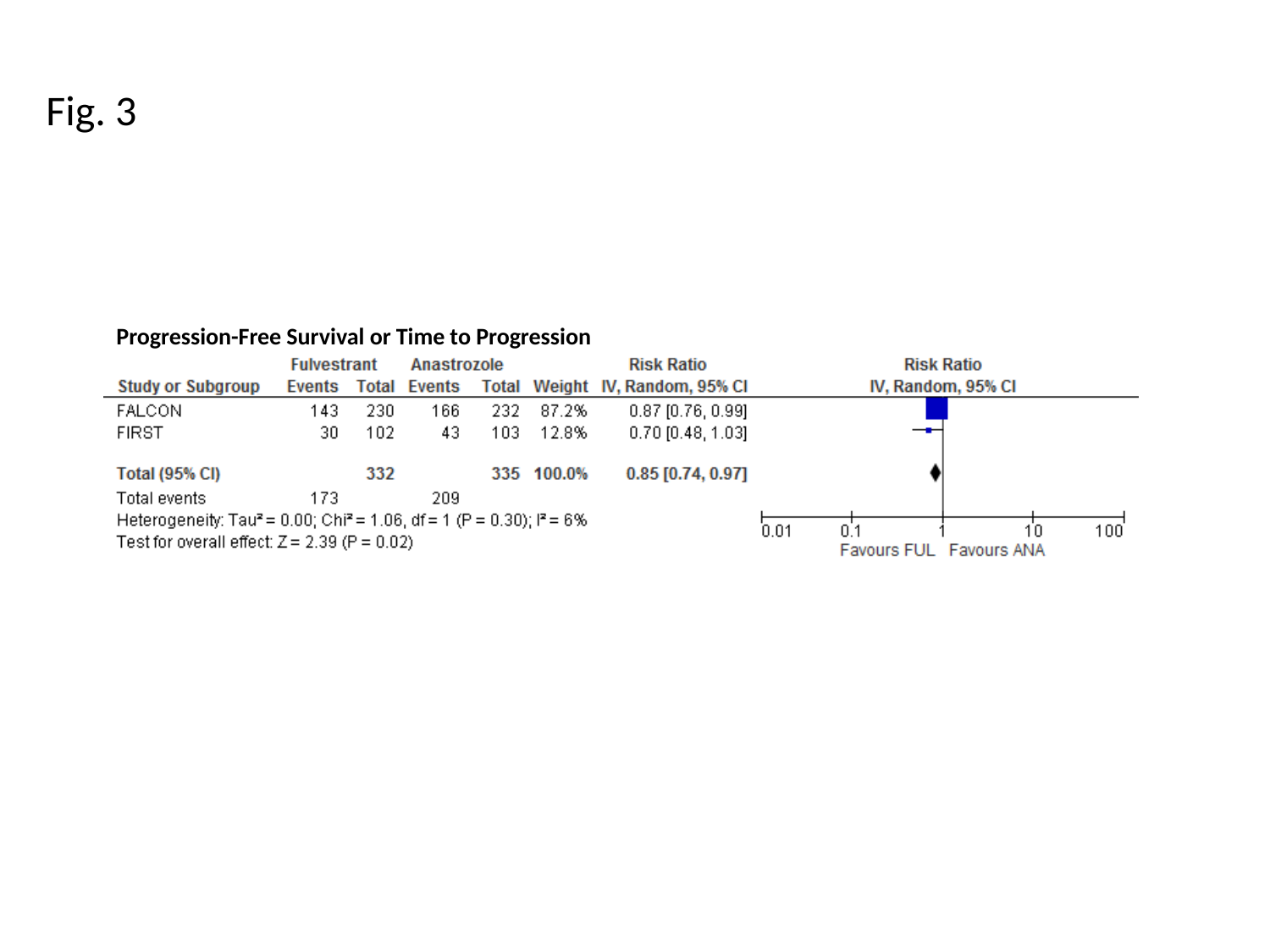

# Fig. 3
Progression-Free Survival or Time to Progression

Supplement: Supplementary file 3 — Supplemental Figure 3. Integrated analysis comparing fulvestrant to anastrozole as first-line endocrine therapy for metastatic breast cancer in postmenopausal patients. (PPTX 41 kb) [file 12282_2020_1085_MOESM3_ESM.pptx]
