# Supplementary material for: The Japanese Breast Cancer Society Clinical Practice Guidelines for systemic treatment of breast cancer, 2018 edition
Source: Breast Cancer. 2020 Apr 2;27(3):322–31. doi: 10.1007/s12282-020-01085-0 (PMC8062371; doi:10.1007/s12282-020-01085-0)
Supplement: Supplementary file 4 — Supplemental Figure 4. Meta-analysis comparing anthracycline-containing regimens with regimens that use both anthracyclines and taxanes (such as CMF), as first-line chemotherapy for patients with metastatic breast cancer. (a) Overall survival, (b) overall response rate. (PPTX 123 kb) [file 12282_2020_1085_MOESM4_ESM.pptx]

## Slide 1
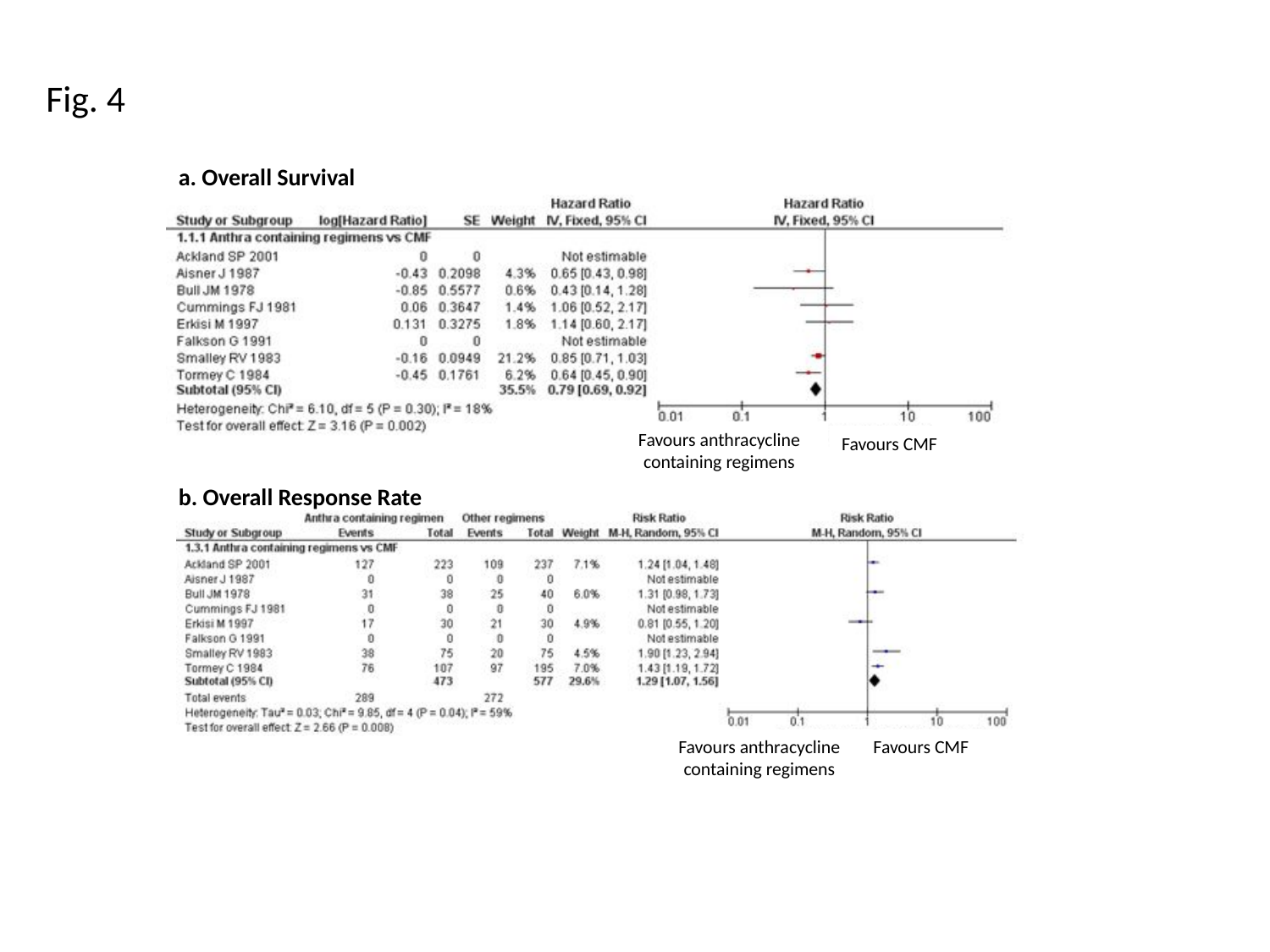

# Fig. 4
a. Overall Survival
Favours anthracycline containing regimens
Favours CMF
b. Overall Response Rate
Favours anthracycline containing regimens
Favours CMF
