# Supplementary material for: The Japanese Breast Cancer Society Clinical Practice Guidelines for systemic treatment of breast cancer, 2018 edition
Source: Breast Cancer. 2020 Apr 2;27(3):322–31. doi: 10.1007/s12282-020-01085-0 (PMC8062371; doi:10.1007/s12282-020-01085-0)
Supplement: Supplementary file 6 — Supplemental Figure 5. Meta-analysis comparing taxane monotherapy with anthracycline-containing regimens as first-line chemotherapy for patients with metastatic breast cancer (c) overall response rate (PPTX 81 kb) [file 12282_2020_1085_MOESM6_ESM.pptx]

## Slide 1
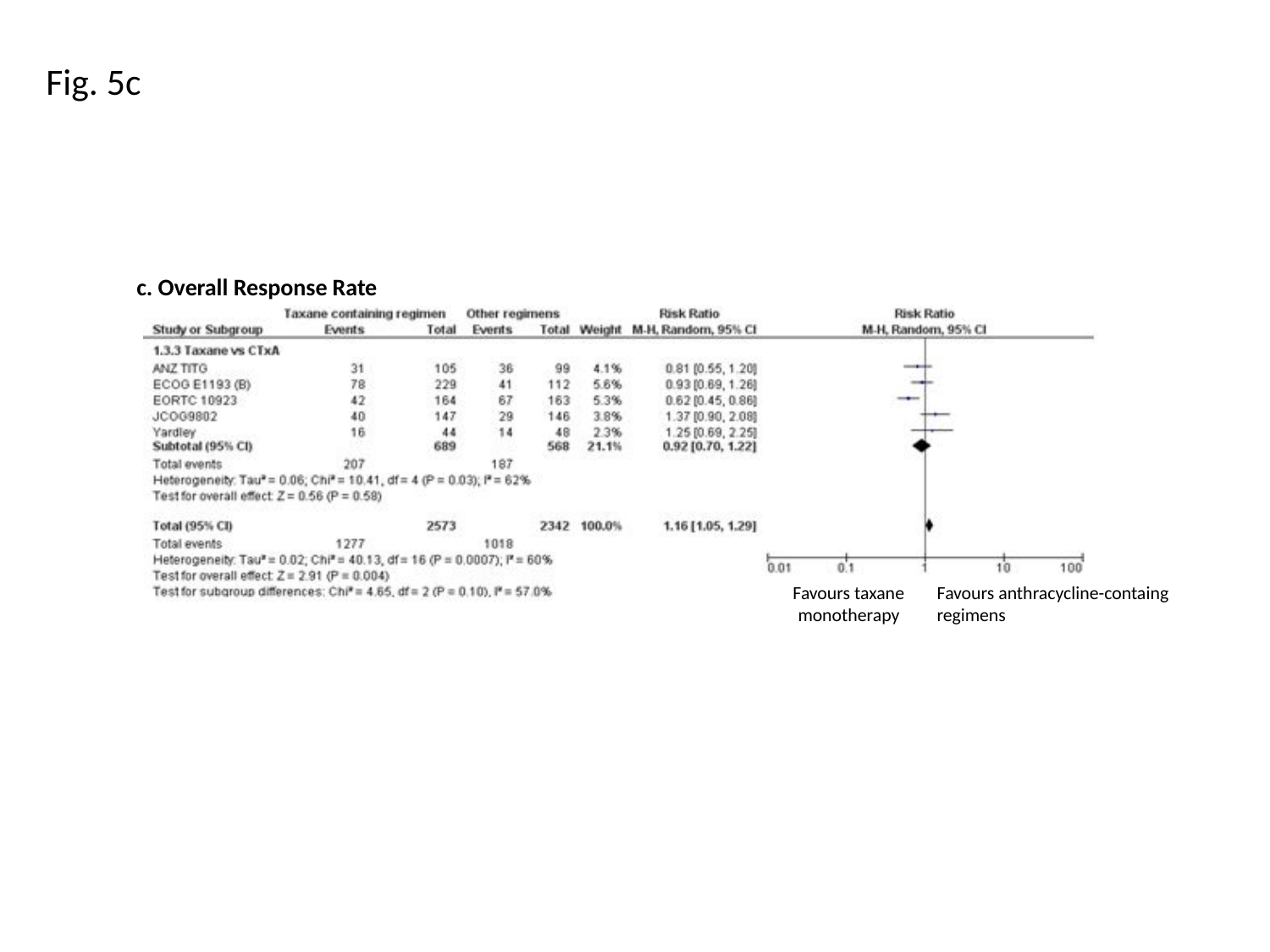

# Fig. 5c
c. Overall Response Rate
Favours taxane monotherapy
Favours anthracycline-containg regimens
