# Supplementary material for: The Japanese Breast Cancer Society Clinical Practice Guidelines for systemic treatment of breast cancer, 2018 edition
Source: Breast Cancer. 2020 Apr 2;27(3):322–31. doi: 10.1007/s12282-020-01085-0 (PMC8062371; doi:10.1007/s12282-020-01085-0)
Supplement: Supplementary file 7 — Supplemental Figure 6. Meta-analysis comparing taxane + trastuzumab with taxane-only as first-line therapy for patients with HER2-positive metastatic breast cancer. (a) Overall survival, (b) progression-free survival. (PPTX 266 kb) [file 12282_2020_1085_MOESM7_ESM.pptx]

## Slide 1
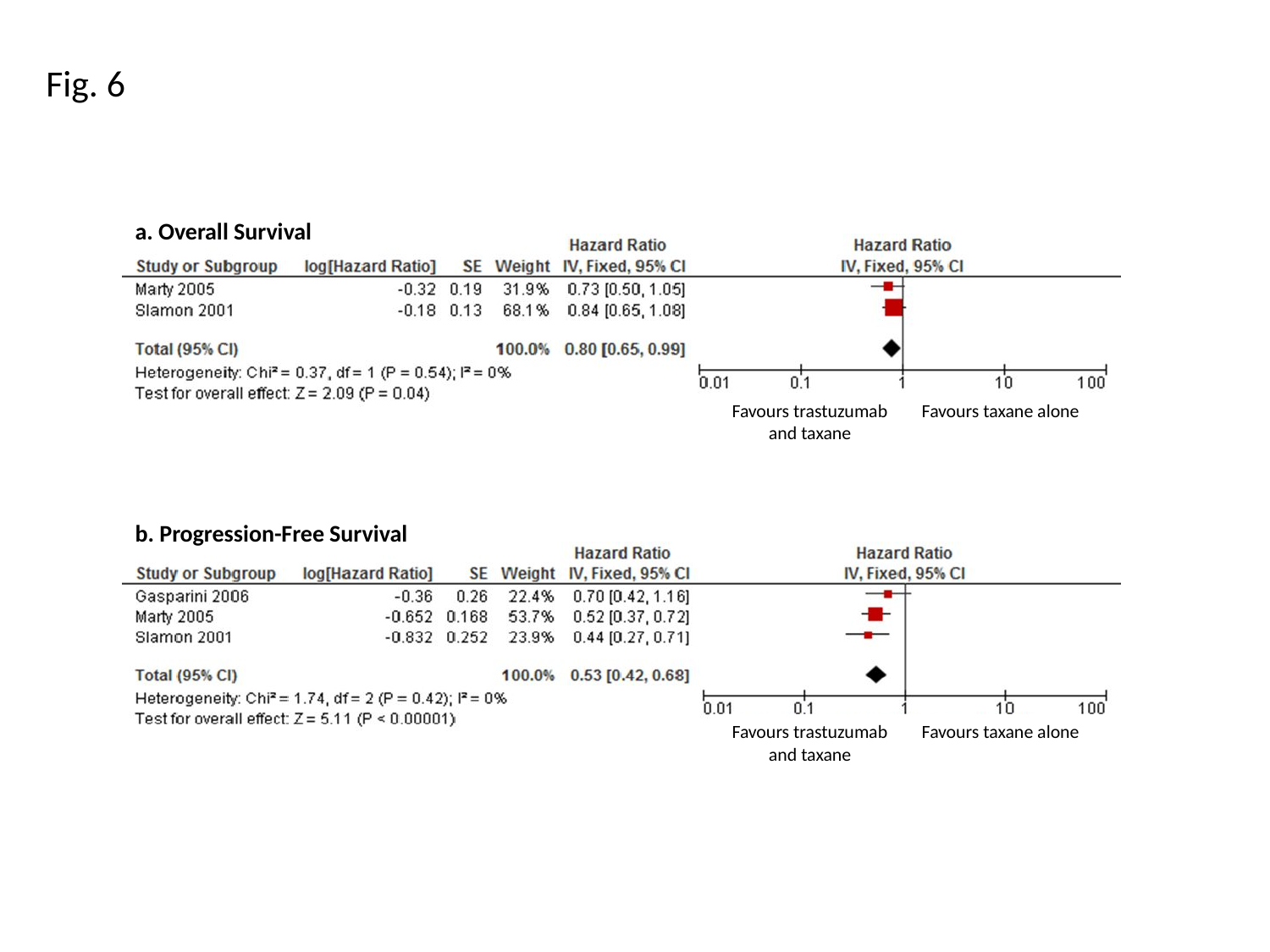

# Fig. 6
a. Overall Survival
Favours trastuzumab and taxane
Favours taxane alone
b. Progression-Free Survival
Favours trastuzumab and taxane
Favours taxane alone
